# Understanding Consumer Online Impulse Buying in Live Streaming E-Commerce: A Stimulus-Organism-Response Framework

**DOI:** 10.3390/ijerph19074378

**Published:** 2022-04-06

**Authors:** Mingwei Li, Qingjin Wang, Ying Cao

**Affiliations:** 1 Business School, Qingdao University, Qingdao 266061, China; wangqingjin2005@126.com; 2 Economics School, Qingdao University, Qingdao 266061, China; caoying@qdu.edu.cn

**Keywords:** impulse buying, live streaming e-commerce, social presence, SOR framework

## Abstract

With the proliferation of live streaming, there is evidence that online impulse buying is becoming an emerging phenomenon. Although many studies have investigated impulse buying in the context of offline shopping and business-to-consumer e-commerce, online impulse buying in live streaming has attracted little attention. In this study, we aim to explore the effect of social presence in live streaming on customer impulse buying based on the stimulus–organism–response framework. The research model presented here identifies pleasure and arousal as the mediation of impulse buying in live streaming. We use the AMOST and IBM SPSS PROCESS software to estimate our model based on data at the minute level from 189 customers, who watched live streaming in the past three months. The results suggest that the social presence of the broadcaster and the social presence of the live streamer positively affect impulse buying directly and indirectly via pleasure and arousal, promoting consumer online impulse buying in live streaming, but the social presence of the viewers has no significant effect on pleasure and arousal. For practice, our results can help policymakers and operators of the live streaming platform alleviate impulse buying in the digital world.

## 1. Introduction

The prevalence of live streaming has driven a boom in e-commerce activities, namely live streaming e-commerce [1,2,3,4]. This new e-commerce mode uses live streaming to engage customers on e-commerce platforms, where broadcasters leverage the new medium as a direct source for online sales [5]. According to a report by Deloitte [6], China owns the world’s largest live streaming market, which reached USD 4.4 billion in 2018. Especially, Alibaba’s Taobao Marketplace, one of the e-commerce giants in China, generated more than USD 15.1 billion in gross merchandise volume through live streaming in 2018. Several factors explain the rapid development of live-streaming e-commerce in the past years. One factor is that the advanced communication infrastructure such as the 5G network facilitates the broadcasting of high-quality live videos. Another factor is that the isolation caused by the COVID-19 coronavirus urgency has further led to the expansion of live-streaming e-commerce because live streaming as a digital tool empowers vendors and customers around the world to be connected [7,8].

In typical live streaming e-commerce, the broadcaster can create and deliver real-time video to the customers. The broadcaster can engage potential audiences to join the live stream to consume the content. For example, the broadcasters can talk about the last beauty trend, introduce the product, and try on different items for the viewers. During the content consumption process, the viewers can also ask questions about the products, chat with the broadcaster and other viewers via text, and even send a virtual gift to their appreciated broadcasters. Compared with the traditional web-based e-commerce or social e-commerce, live streaming e-commerce can not only allow the customer to become closer to the products but also to hear the broadcaster describe how the product feels and watch the effect of the broadcaster try it on, thus fostering a more authentic and interactive online shopping experience [9,10]. Many customers could not help buying the product when watching live streaming e-commerce.

Impulse buying refers to an unplanned purchase, the result of exposure to a stimulus, and deciding to purchase on the spot [11,12]. According to a report from Ishita [13], more than 80% of younger customers conduct impulse buying online, especially for the product categories of groceries and household staples. Compared with web-based e-commerce, live streaming can foster more authenticity and interactivity during online shopping, thus promoting more customers to buy impulsively [10,14]. For example, Jiaqi Li, one of the Chinese live-streaming broadcasters in Taobao Live, has set a record of selling more than 15,000 lipsticks in 5 min during live streaming e-commerce. As one of Li’s fans said in an interview, “His broadcast with full of passion attracts me the most and arouse my enthusiasm to buy products [15]. ”Another fan said, “… I don’t understand why I wanted to purchase the product within the first 30 s, and I was even worried that it was out of stock [15].” This study attempt to understand which factors influence consumer online impulse buying in live streaming is a relatively new viral e-commerce phenomenon.

During the last decade, adequate research attention has been paid to online impulse buying. Current findings in the context of web-based e-commerce and social e-commerce context may not be suitable for impulse buying in live streaming e-commerce [16,17,18]. Compared to these modes, live streaming e-commerce can foster a more authentic and interactive shopping experience and bridge the distance between customers and presented products [10,14]. The concept of social presence can capture the virtual shopping experience brought by live streaming e-commerce [19,20,21]. However, some studies only investigated trust, flow state, interactive activities, attractiveness and expertise of the live streamer, and the live streaming purchase convenience on impulse buying in live streaming e-commerce. It is still unclear how social presence in live streaming influences impulse buying through an emotional mechanism, considering that the emotional state is the main driver of the impulse behavior.

To address this question, we use the stimulus–organism–response (SOR) framework to conceptualize our theoretical model because the SOR framework provides a suitable lens to understanding the mechanism for impulse buying via social presence in live streaming. The SOR framework of environmental psychology was proposed by Mehrabian and Russell [22]. The SOR framework suggests that some environmental aspects resulted in certain behavioral outcomes through provoking an individual’s emotional and cognitive conditions. Therefore, it was widely used in the research of consumer behaviors in the marketing and information system disciplines [1]. The SOR framework consists of three components: stimulus, organism, and response. In our research, the social presence that captures the overall virtual experience within live streaming e-commerce is the stimulus in our model. Some research used a single dimensional to conceptualize social presence. However, Song et al. [23] and Lu et al. [24] suggest that the unidimensional conceptualization of social presence is oversimplistic in the context of computer-based communication because individuals not only communicate with the medium but also interact with other participants (e.g., the sellers and other customers). Following the paper by Song et al. [23], we used three dimensions to conceptualize social presence in live streaming: the social presence of the live streaming, the social presence of the broadcaster, and the social presence of the viewers. The organism is the customers’ affective and cognitive conditions. In our research, impulsive buying behavior is characterized by consumers’ emotional activation rather than cognitive factors [25]. Therefore, the emotional factors of pleasure and arousal are the organism. Specifically, pleasure refers to the degree to which individuals feel happy or satisfied, and arousal refers to the degree of stimulation caused by an atmosphere [22]. Third, impulse buying in live streaming is the response. Impulse buying refers to “a sudden and immediate purchase with no pre-shopping intentions either to buy the specific product category or to fulfill a specific buying task. The behavior occurs after experiencing an urge to buy and it tends to be spontaneous and without a lot of reflection” (i.e., it is “impulsive”) [11,26].

Based on the SOR framework, we construct and empirically test a structural model that examines impulse buying as a behavioral response caused by the social presence in live streaming e-commerce by using sampling data of 189 consumers from Taobao Live in China. Our study not only contributes to the literature on live streaming e-commerce and social presence theory but also provides practical insights for policymakers and live streaming operators to alleviate impulse buying in live streaming e-commerce.

The remainder of the paper is organized as follows. First, we review three streams of related literature. Second, based on current studies, we propose a theoretical model and our hypotheses. Third, we introduce our research context, sampling and data collection, and measure items. Fourth, we empirically test the research model and hypotheses, followed by a presentation of the research results. Finally, this paper concludes with the theoretical and practical implications and limitations that give rise to further research. 

## 2. Related Literature

### 2.1. Live Streaming E-Commerce

Live streaming is a type of user-generated content [1]. It allows people to stream live content, such as singing, dancing, or playing video games [2]. In the beginning, the live streaming platforms are mainly focused on gaming and entertainment. With its development, it also boosts its fusion with market campaigns, thus the rise in live streaming e-commerce [3]. With the evolution of live streaming e-commerce, there are two main types: e-commerce sites with live streaming features, and social network platforms with e-commerce activities [27]. The former indicates that live streaming features as an alternative way to present products or services that are embedded into the e-commerce website [1,3]. Some examples include Amazon Live and Taobao Live, and Taobao live is one of the e-commerce giants in China, while the latter indicates that e-commerce activities are embedded into social network platforms or short video platforms [28], for example, Facebook Live or YouTube Live. Some Chinese short video platforms, such as Douyin, also incorporate e-commerce activities into their platform. 

Live streaming e-commerce is more likely to induce customers’ impulse buying behaviors than traditional website e-commerce for the following reasons. First, in the traditional e-commerce website, interactive activities mainly occur between the customer and the website features [29]. However, with the advantage of digital technologies, broadcasters can upload real-time video content and present the products from different perspectives in live streaming e-commerce. The traditional customer–website interaction has shifted to the interactive activities between broadcasters and customers or among different customers in live streaming e-commerce [9,29]. Second, live streaming e-commerce can foster a better sociable and more authentic experience for customers than a traditional e-commerce website [10,30]. Broadcasters in live streaming can present and provide detailed information of the products or try on the products (e.g., cosmetics and clothes). The customers can also receive real-time feedback from broadcasters in live streaming e-commerce. Conversely, viewers in the live stream can also interact with each other to share shopping experiences by sending texts in the chatbox. Therefore, live streaming e-commerce can foster a more interactive, authentic, and visual shopping experience, which attracts more potential customers and improves the customer’s impulse buying rate.

With the development of live streaming e-commerce, growing attention has been paid to this emerging topic. However, the current research efforts have mainly focused on customer engagement and purchase intention in live streaming e-commerce [3,10,30]; impulse buying as a large portion of sales in e-commerce gains relatively less attention compared with current studies. In the following section, we review the literature related to online impulse buying. 

### 2.2. Online Impulse Buying

Online impulse buying can be defined as “a purchase that is unplanned, the result of an exposure to a stimulus, and decided on the spot” [12]. Some recent studies have provided a more extensive conceptualization in that impulse buying is “a process–outcome mechanism within the domain of an individual–psychological approach that occurs when a consumer experiences a sudden, often persistent urge to buy something immediately” [31,32]. Such impulse buying occurs after experiencing an urge to buy and may stimulate emotional conflict [11,26]. Impulse buying can be further divided into four different types: pure impulse buying, reminder impulse buying, suggestion impulse buying, and planned impulse buying [33,34]. Most current studies adopted the pure impulse buying proposed by Beatty and Ferrell [11], which was usually measured using survey questionnaires. In our research, we used three items to operationalize online impulse buying.

With the progress of live streaming e-commerce and its application, online impulse buying is common among customers [31,35,36]. First, previous live streaming studies have mainly focused on factors that influence customer impulse buying, such as social presence, customer trust, flow state, and IT affordance. For example, Ming et al. [14] investigated how presence influences customer impulse buying in the context of live streaming commerce. They found that social presence influences customer flow state and trust, thus causing impulse buying. Sun et al. [10] investigated how live streaming influences customers’ purchase intention in social commerce. They found that visibility affordance, meta-voicing affordance, and guidance shopping affordance can positively influence customer purchase intention through live streaming engagement. Second, interactive activities in live streaming induce consumers to make a purchase [29]. For example, Wongkitrungrueng et al. [37] indicated that the interactive activities among live broadcasters and audiences and the authentic presentation of products can easily induce customer buying behavior. Third, the current studies also investigated the attractiveness of the live streamer, the expertise of the live streamer, and the live streaming purchase convenience on impulse buying responses [38,39].

### 2.3. Social Presence

Social presence refers to the “degree of salience of the other person in the interaction and the consequent salience of the interpersonal relationships” [40]. The concept of social presence originates from the field of social psychology and describes the degree to which individuals perceive the presence of participation from the use of telecommunications [41]. It was first used in the settings of mediated communication and then extended to information and communication technology (ICT) research to explain the social aspect of technology [42]. With the internet becoming a critical retailing channel, the concept of social presence, as a virtual experience, has been widely used to study customer behavior in an ICT-enabled virtual environment, such as ICT-mediated communication, online e-commerce, social media e-commerce, and live streaming e-commerce [19,23,24].

Social presence plays an important role in the online shopping context. Currently, social presence has often measured the warmth of media or human feeling of sociability from ICT [24]. However, this one-dimensional conceptualization of social presence might not be suitable in live streaming e-commerce, because customers not only use live streaming features to interact with the broadcaster but also to interact with other viewers in the virtual room. Thus, a multi-dimensional conceptualization may be more suitable in the context of live streaming e-commerce. This multi-dimension conceptualization aligns well with the one dimension for considering social presence as the subjective quality of the medium, which makes the interactions more social and salient. Therefore, we conceptualize the social presence in live streaming e-commerce from three dimensions: the social presence of the broadcaster, the social presence of viewers, and the social presence of live streaming e-commerce.

The social presence of the broadcaster refers to the extent to which customers perceive the direct interaction with the broadcaster in live streaming [14,24]. The live streaming room is a virtual world, the interaction between broadcasters and viewers transcends time and space. The live broadcasters can display products in detail, interact with the audiences, and offer them personalized service in this virtual space in real time [37]. Therefore, live streaming commerce has a better sense of social presence. For example, Guo et al. [43] used the concept of the broadcasters’ screen presence to investigate the factors that influence customer participation behavior in the context of mobile live streaming. They found that the customer commerce behaviors are determined by broadcasters’ screen presence, especially the facial and hand appearances.

The social presence of viewers refers to the extent to which customers perceive the presence of other customers in the live streaming [14]. The chatbox feature in live streaming e-commerce can also increase the social presence through interaction with other viewers in the virtual room because the interactions among viewers can make online shopping more social. Conversely, consumers can share information about products in the live stream, and word-of-mouth valence from other viewers can play an informative role for customers to better know the products and experience others’ consumption. 

The social presence of live streaming refers to the live streaming’s capability to convey a feeling of human contact, sociability, warmness, and sensitivity [3]. In traditional online shopping, consumers can only see some pictures of the products or text descriptions of the products from sellers [44]. While live streaming e-commerce allows audiences to watch video streams in real time, this shopping experience can enhance the sense of sociality, sensitivity, and human contact through communication using voice. For example, Shen and Khalifa [25] indicated that when the customer experiences a sense of social presence in computer-mediated interaction, the experience will reduce the distance between products and customers, thus fueling impulse buying on the internet. 

### 2.4. Pleasure and Arousal

Mehrabian and Russell [22] indicated that dimensional emotional states can be divided into two dimensions: pleasure/displeasure and arousal/sleepiness. Pleasure refers to the degree to which individuals feel happy, joyful, or satisfied. Arousal refers to the degree to which individuals feel stimulated, excited, or alert [22]. Prior empirical evidence shows that the emotional state is the main driver of impulse behavior [25]. Therefore, the dimensional emotional state has also been widely applied in the online and offline shopping contexts to investigate how environmental and atmospheric cues influence the consumer’s online behaviors. For example, Hsieh et al. [45] investigated the effect of pleasure and arousal in customer-brand relationship building. Liao et al. [46] also indicated that the presentation mode and product type can increase consumers’ pleasure and arousal, inducing impulse buying in the traditional online shopping industry.

## 3. Research Model and Hypothesis

### 3.1. Theoretical Model

**Figure 1:**
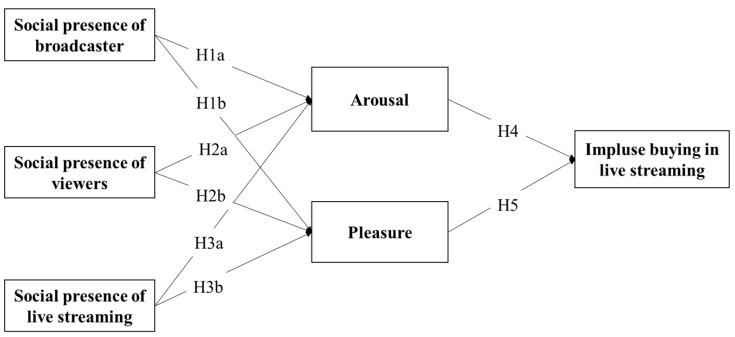
Our research model.

Our research model is shown in Figure 1. Specifically, social presence is an element of the live streaming e-commerce experience and can thus be considered as the stimuli. Impulse buying in live streaming can be considered as the behavioral response. According to the model, stimuli of live streaming e-commerce context can influence individuals’ cognitive and emotional states, which may in turn trigger customer behavioral response. Some research evidence has confirmed that the emotional state is the main driver for impulsive behavior. Thus, we consider two basic emotional states of pleasure and arousal as part of the organism that mediates the behavioral response to the stimuli in live streaming e-commerce.

### 3.2. Hypotheses

In live streaming e-commerce, the broadcaster can introduce and try the products as well as provide personalized services for the audiences [3]. For example, customers can ask for the broadcaster to try on a specific sized T-shirt for her/him, and then she/he can observe the effect of the product. During the consumption process, the customers can also engage with the broadcasters in real time in various ways, such as chatting, sending likes, and tipping [2]. These interactions can enhance the social presence of the broadcaster as they are communicating face-to-face, which will thus increase the customers’ shopping pleasure in the live streaming. Prior studies also report that the stimuli in physical shopping can influence shoppers’ pleasure and arousal [47]. For example, Baker et al. [48] found that a friendly salesperson has a positive effect on customer pleasure and arousal in offline settings. Therefore, we propose the following hypothesis:

**Hypothesis** **1a** **(H1a).** *The social presence of the broadcaster has a positive influence on customers’ arousal in live streaming e-commerce*.

**Hypothesis** **1b** **(H1b).** *The social presence of the broadcaster has a positive influence on customers’ pleasure in live streaming e-commerce*.

In live streaming e-commerce, the broadcaster presents various content to interact with the existing customers and attract new viewers into the virtual showroom. The audiences in the live streaming can also interact with the other viewers using text in the chat channel [3]. Communication among viewers makes online shopping more sociable and sensitive. Moreover, viewers can have a better understanding of the products through other viewers’ word of mouth [49]. This can make customers co-experience shopping in live streaming e-commerce together, thus enhancing customer viewing experience. When customers are immersed in virtual shopping activities, they tend to experience a state of pleasure and arousal. Thus, it is reasonable to expect that customers who are aware of other viewers in live steaming e-commerce are more likely to experience pleasure and arousal.

**Hypothesis** **2a** **(H2a).** *The social presence of viewers has a positive effect on customer arousal in live streaming e-commerce*.

**Hypothesis** **2b** **(H2b).** *The social presence of viewers has a positive effect on customer pleasure in live streaming e-commerce*.

The social presence of live streaming assesses the subjective characteristics of live streaming e-commerce, which reflect the social side of IT-enabled communication. Prior empirical evidence also suggests that the social presence of media communication can enhance the emotions of pleasure and arousal [25]. Some research suggests a strong correlation of social presence to pleasure and arousal in the circumstance of online e-commerce [31]. For example, consumers have a stronger sense of social presence when they are interacting with a computer “tutor” on an e-commerce website [25,50]. The more warm and friendly the customers feel from the computer “tutor”, the more emotionally satisfied they feel. 

Compared with online e-commerce websites, live streaming e-commerce has more human touch and sociability with the broadcasters and other viewers [3,5]. Thus, the circumstance of live streaming e-commerce will make the customers feel more natural and sociable [4,14]. In live streaming, the broadcaster delivers content or product to the audiences in the online space in real time. Viewers can join the live streaming to consume the content or purchase the products. This interactive experience makes customers feel that they were physically present during the live streaming e-commerce, thus increasing customer emotions of pleasure and arousal. Therefore, we propose the following hypothesis.

**Hypothesis** **3a** **(H3a).** *The social presence of live streaming has a positive influence on customer arousal in live streaming e-commerce*.

**Hypothesis** **3b** **(H3b).** *The social presence of live streaming has a positive influence on customer pleasure in live streaming e-commerce*.

According to Mehrabian and Russell’s [22] model, the emotional reactions (i.e., pleasure and arousal) to the environment determine the individual response (i.e., impulse buying in live streaming e-commerce). First, pleasure can serve as the motivation for impulse buying in live streaming e-commerce [1]. Shen et al. [25] indicated that customers who feel pleasure when they are shopping tend to use this affective state to make a judgment. In other words, the customers who are in a pleasant state are more likely to conduct impulse buying in live streaming e-commerce. Second, if customers are in a pleasant context, arousal predicts the approach behaviors [1]. The aroused customer will allocate more attention to the live streaming and will tend to continue browsing the broadcaster-recommended products and services [10]. The extended exploring experience may enhance the impulse buying behaviors in live streaming. In short, the effect of pleasure and arousal on impulse buying behaviors has been investigated in both offline shopping contexts and online contexts (e.g., Internet-based e-commerce). Prior empirical evidence shows that pleasure and arousal as customers’ affective reactions can trigger customer impulse buying [51]. We suggest that the same logic can be extended into the circumstance of live streaming e-commerce.

**Hypothesis** **4** **(H4).** *Consumer arousal is positively related to impulsive buying in live streaming e-commerce*.

**Hypothesis** **5** **(H5).** *Consumer pleasure is positively related to impulsive buying in live streaming e-commerce*.

## 4. Materials and Methods

### 4.1. Study Context

In this research, we considered Taobao Live, one of the major e-commerce platforms run by Alibaba in China, as our research context. Taobao Live is the live streaming service integrated into Taobao’s e-commerce App. In 2018, Taobao Live provided over 3000 live broadcasts under the guidance of its live streamers [3]. Taobao Live is different from other Chinese live streaming platforms (e.g., Douyin Live and Kuaishou Live) in that Taobao Live embeds into Alibaba’s e-commerce platform, while Douyin Live and Kuaishou Live embed e-commerce business into the live streaming platform [3].

**Figure 2:**
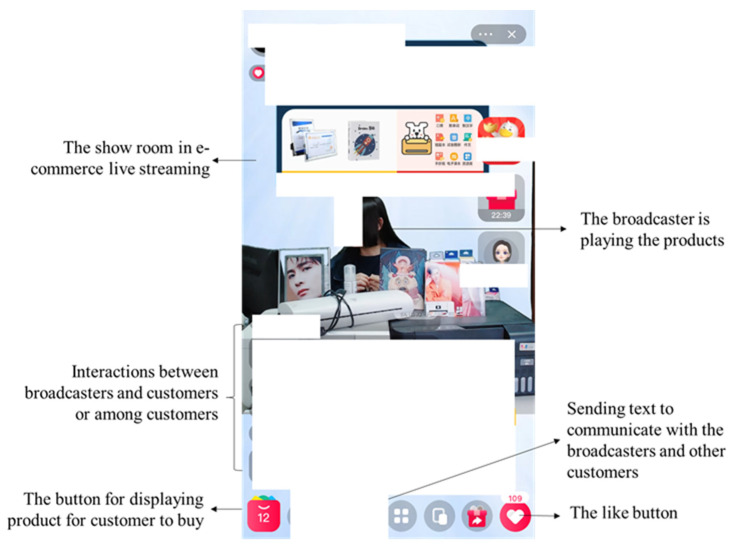
Screenshots of live streaming e-commerce on Taobao Live.

In typical live streaming e-commerce, broadcasters can build the video streams and display and introduce the products (e.g., clothes and cosmetics) for customers. Customers can interact with the broadcasters and other customers using text. Figure 2 provides screenshots of a typical ongoing live streaming e-commerce in Taobao Live from the customers’ view. This enhanced interactive sociability and contact in live streaming e-commerce can easily trigger customers’ impulsive buying behaviors.

### 4.2. Sampling and Data Collection

An online survey was conducted to collect data to test our hypotheses. Specifically, we used the Wenjuanxing website (www.wjx.cn, accessed on 17 February 2022) to develop the questionnaire and chose a professional data collection service offered by the website to collect our data. Wenjuanxing has access to a sample database of 26 million Chinese respondents [52] and can help to select the live streaming e-commerce customers. Since the survey was conducted in China, the original English items were translated into Mandarin Chinese. To ensure consistency between these two versions, a backward translation was conducted [53]. Our questionnaire included one screen question, four customer demographic information items (i.e., age, gender, income, and watching frequency), and nineteen measurement items for six variables. To ensure the suitability of all the respondents, a screen question regarding if live streaming e-commerce (such as Taobao Live) was watched within the past month was added. Only those respondents who answered “yes” had access to the following questions. Then, the respondents were instructed to answer the following questions by recalling the last live streaming e-commerce experience. In total, 243 responses were collected and 54 of these responses were deleted for missing data. The final data for analysis included 189 respondents, and the response rate was 77.78%.

*To assess for non-response bias, we compared the demographic variables of the early (i.e., the first 50 respondents) and late (i.e., the last 50 respondents) waves of the completed surveys. The Mann–Whitney test shows that the two groups do not differ in age (p = 0.81), gender (p = 0.69), or income (p = 0.52). These results suggest that the nonresponse bias is not a concern in this study.4.3. Measure Items*

The multiple-item scales used in our study are designed based on the relevant literature, and minor changes were made to fit the context of live streaming e-commerce (see Table 1). Social presence is measured using three dimensions—the social presence of the broadcaster, the social presence of the viewers, and the social presence of live streaming e-commerce. Measurement items for the social presence of the broadcaster are adapted from [14,20,52]. Measurement items for the social presence of the viewer and the social presence of the live streaming are adapted from [24,54]. Measurement items for pleasure and arousal are adapted from [22,25]. Measurement items for pleasure and arousal are adapted from [22,25]. Measurement items for impulse buying in live streaming e-commerce are adapted from [47,55]. All constructs were measured using multi-item reflective indicators on a 5-point Likert scale ranging from “1 = strongly disagree” to “5 = strongly agree”.

## 5. Results

### 5.1. Sample Characteristics

The demographic information of surveyed respondents is shown in Table 2. Of the respondents, the majority are females (53.44%), in the age bracket of 26 to 35 years old (58.20%), with an income less than 2000 yuan (47.09%), watching live streaming e-commerce three to five times in the past three months (43.92%).

As our data were collected from a cross-sectional survey, common method bias (CMB) may exist. Following [56], we used a post hoc method to analyze CMB. Specifically, we conducted Harman’s one-factor test on the 18 items to assess for CMB. The result shows that the most observed variance explained by one factor is 23.605%, less than 40%. Therefore, the CMB is not an issue in this study.

### 5.2. Measurement Model

To examine the reliability and validity of measures in our model, we conducted three types of necessary tests: internal consistency, convergent validity, and discriminant validity. First, we used the composite reliabilities [45] to assess the internal consistency of the items. As seen in Table 3, the value of all CR is above the minimum level of 0.7. All the item loading was above the reliability threshold level of 0.7. These results show that the data have internal consistency. Second, we used [57] criterion of average variance extracted (AVE) to assess the convergent validity of each construct. The results show that the AVE of all constructs ranges from 0.50 to 0.67, thus exceeding the minimum required of 0.5. Third, we used the inter-construct correlations to assess discriminant validity. As shown in Table 4, the square root of the AVE of each construct is higher than the correlation between constructs, and all correlations are less than the square root of AVE, which establishes discriminant validity. The visual inspection of loadings for this study and cross-loadings further confirm that all constructs had good discriminant validity (see Table A1 in Appendix A).

### 5.3. Structural Model

We used AMOST to estimate the path coefficient and the significance of the hypothesized relationship. In AMOST, we used the fit criterion of χ²/df, GFI, RMR, CFI, NFI, and NNFI to evaluate the structure model. Most of the criterions exceed the stipulated threshold except for PNFI: χ²/df = 1.197, GFI = 0.924, RMSEA= 0.032, RMR = 0.031, CFI= 0.984, NFI = 0.913, AGFI = 0.895, and NNFI = 0.981. AGFI is below its threshold (0.9), but it is common that not all fit criteria are perfect in the structural model [24,58]. The structure model results show a good explanatory power for impulse buying in live streaming e-commerce (R^2^ = 0.703). The presence of the broadcaster, the presence of the viewer, and the presence of the live streaming proved to be effective in creating customers’ pleasure (R^2^ = 0.518) and arousal (R^2^ = 0.658). All these results provide strong support for our structure model.

**Figure 3:**
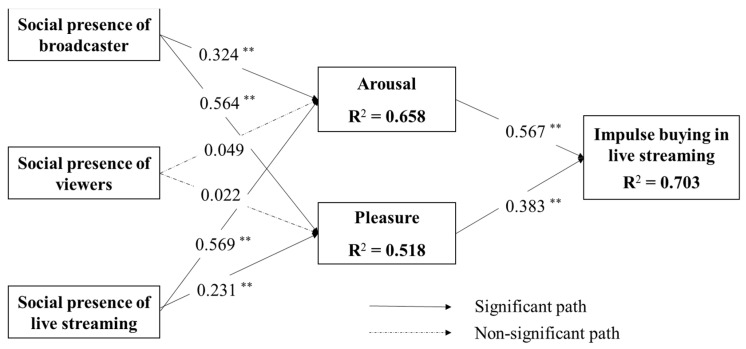
Results of the structure mode. Note: ** *p* < 0.01.

The estimated path coefficients, related hypotheses, and variances explained are shown in Table 5 and Figure 3. The results show that six out of the eight hypotheses (i.e., H1a, H1b, H3a, H3b, H4, and H5) are supported for the p value less than 0.05. Specifically, SPOB has a direct and positive relationship on arousal (β = 0.324, t = 3.652, *p* < 0.01) and pleasure (β = 0.564, t = 6.297, *p* < 0.01). These findings support H1a and H1b. SPOV has no significant effect on arousal (β = 0.049, t = 0.526, *p* = 0.599) and pleasure (β = 0.022, t = 0.242, *p* = 0.808). As a result, the findings do not support H2a and H2b. SPOLS has a direct and positive relationship on arousal (β = 0.569, t = 4.694, *p* < 0.01) and pleasure (β = 0.231, t = 2.254, *p* < 0.01). These findings support H3a and H3b. The coefficients of social presence of live streaming on both arousal and pleasure are stronger than the coefficients of social presence of the broadcaster on arousal and pleasure. 

Arousal has a direct and positive relationship on IB (β = 0.567, t = 5.949, *p* < 0.01). Pleasure has a direct and positive relationship on IB (β = 0.383, t = 4.646, *p* < 0.01). These findings support H4 and H5. These results are consistent with prior studies; pleasure and arousal were the critical determinants of impulse buying [16,43]. The results mean that the emotional experience of pleasure and arousal in live streaming e-commerce has strong implications for action tendency. The coefficient of pleasure on impulse buying in live streaming is stronger than the coefficients of arousal on impulse buying.

### 5.4. Additional Analysis

We further examined the role of pleasure and arousal in mediating the effect of social presence on (i.e., the social presence of the broadcaster, the social presence of the viewers, and the social presence of the live streaming) impulse buying in live streaming e-commerce. We used PROCESS in SPSS [59,60] to conduct the multiple mediation analysis. Table 6 shows the results of the mediating effects. The indirect effect of the social presence of broadcaster and social presence of live streaming on impulse buying in live streaming e-commerce through pleasure and arousal is significant because the intervals of the bootstrap 95% confidence interval (CI) do not include 0. The direct effects of SPOB and SPOLS on impulse buying are significant; thus, pleasure and arousal partially mediated the effects of SPOB and SPOLS on impulse buying. However, the indirect effect of social presence of viewers on impulse buying in live streaming e-commerce through pleasure and arousal is not significant because the intervals of the bootstrap 95% confidence interval (CI) include 0.

## 6. Discussion

The objective of this study was to understand consumer impulse buying in live streaming. Using the SOR framework as our theoretical lens, we investigated the role of social presence (stimulus) in impulse buying (response) in live streaming e-commerce by considering customer pleasure and arousal (organism). 

The findings confirm that the social presence of the broadcaster and the social presence of the broadcaster of the live streaming can predict customer arousal and pleasure. However, our findings suggested that there is no direct effect of the social presence of the viewers on impulse buying in live streaming e-commerce. One plausible explanation is that customers may suspect the motives of revealing the social cues or the authenticity of those social cues from other viewers; thus, these social cues from other viewers are not used by the customer to make purchase decisions [24]. Another reasonable explanation is that most products sold in live streaming e-commerce are experience products (e.g., cosmetics and clothes), and the information provided by other viewers is not credible until they experience the products [1].

Furthermore, consistent with the current literature in both relational marketing and information systems [25,31], impulse buying in live streaming e-commerce can be predicted by customer arousal and pleasure. Through the multiple mediation analysis, we found that the social presence of viewers can only affect impulse buying indirectly via pleasure and arousal. In contrast, the social presence of the broadcaster and the social presence of the live streaming can affect impulse buying both directly and indirectly via pleasure and arousal. These findings have important theoretical and practical implications. 

### 6.1. Theoretical Implications

The study provides several theoretical implications. First, we contribute to the literature on customers’ well-being in the pandemic period by investigating the impulse buying behaviors in the context of live streaming e-commerce. Impulse buying behaviors are commonplace in live streaming e-commerce all over the world. Moser et al. [61] indicated that impulse buying behaviors have many negative impacts, such as feelings of guilt and regret and financial strain. However, with live streaming boosting its fusion with market campaigns, few studies have investigated customer impulse buying in live streaming e-commerce [2,30]. We closed the gap by investigating how three dimensions of social presences affect customer emotional states of pleasure and arousal, thus leading to impulse buying in live streaming e-commerce.

Second, this study contributes to the social presence theory by identifying a three-dimensional social presence in live streaming e-commerce: the social presence of broadcaster, the social presence of viewers, and the social presence of live streaming. Impulse buying in live streaming is typical IT-related behavior, and social presence is one of the critical design principles for customers’ IT-related behavior. The prior studies mainly examined the role of one or two dimensions of social presence (e.g., social presence and telepresence) in IT-related behaviors [24,41,42]. In our study, we conceptualized the social presence in live streaming from three dimensions of broadcaster, viewer, and live streaming platform and investigated how they affect customers’ impulse buying separately. We can examine the influence of the three dimensions of social presence on customer emotions (i.e., pleasure and arousal), thus affecting customer impulse buying in live streaming e-commerce. Conversely, a multi-dimension of social presence in live streaming can help us understand which antecedent factors affect different social presence dimensions. 

Third, our study sheds light on the formation mechanism of impulse buying in live streaming e-commerce by investigating the customer’s emotional state as a mediator. Pleasure and arousal (as a set of emotions) as two key mediating variables have been fruitful in explaining experiential shopping behaviors. Prior studies focused on the effect of specific stimuli such as website quality on customer impulse buying [25,35,36]. In our research, we further extended the use of pleasure and arousal as two key mediating variables by integrating the multi-dimensions of social presence in live streaming e-commerce (i.e., the social presence of broadcaster, the social presence of viewers, and the social presence of live streaming). It can allow us to capture the overall virtual shopping experience brought about by live streaming e-commerce, and it also can show the dimension of social presence as having a significant influence on customers’ emotional state.

### 6.2. Practical Implications

This study also presents important practical implications. First, in the context of COVID-19 emergence, investment in live streaming e-commerce has grown rapidly [62]. Live streaming empowers the business owner to promote their product virtually face-to-face with an interested customer, thus leading to serious impulse buying in live streaming e-commerce. Our findings suggest that the social presence of the broadcaster and the social presence of the live stream are effective in improving customer pleasure and arousal, thus stimulating online impulse buying in the live stream. Therefore, a practical implication for policymakers and operators of the live streaming platform is to select and design specific features to reduce the social presence of the live stream. Moreover, the policymakers and operators of the live streaming platform should strengthen the supervision of broadcasters and should not allow broadcasters to send persuasive information to deceive or mislead consumers during the live stream.

Second, although the social presence of viewers seems to be a weaker prediction for customer pleasure and arousal, this does not mean that viewer presence does not matter for online impulse buying in live streaming. Policymakers and operators of the live streaming platform can limit the number of viewers in the live stream to reduce the social presence of other viewers. Conversely, policymakers and operators should pay more managerial effort to prevent these capper consumers from spreading false product information during live streaming.

Third, the findings show that the customers’ pleasure and arousal play a mediate role between the three dimensions of social presence and impulse buying in live streaming. Therefore, a practical implication for operators of the live streaming platform is to design live streaming features (e.g., soft music and pictures) to alleviate customers’ excited emotional state when they are shopping online. At the same time, consumers should warn themselves not to engage in live streaming e-commerce when they are in a pleasant or stimulated emotional state.

## 7. Conclusions

In conclusion, the main contributions of the study are three-fold. First, during COVID-19, the offline commerce operating modes were seriously interrupted by the emergency. Live streaming has empowered vendors to reach a large audience and to promote their products virtually face-to-face with interested buyers, leading to serious online impulse buying behavior. Based on the SOR framework, we attempted to understand customer impulse buying in live streaming, a relatively new e-commerce phenomenon. Second, we proposed a multi-dimensional conceptualization of social presence in live streaming to overcome the limitation of the one-dimensional social presence construct. Third, we highlighted the importance of three social presence factors in live streaming e-commerce that induce impulse buying via customers’ emotional state (i.e., pleasure and arousal). This study has some limitations. First, the research context is Chinese live streaming e-commerce, which may restrict the external validity of our research findings regarding cultural differences. Future studies can further consider a sample of live streaming users in other countries to examine the external validity of our findings. Second, we are not restricted to a specific market (e.g., clothes or cosmetics) in live streaming e-commerce. It is an empirical question to whether our findings are replicable in a different market. Future studies should modify and apply the current research model to a specific product market. Third, in our research, we focused on social presence in live streaming to conceptualize customers’ virtual experience, which is relevant to inducing impulse buying. Future studies can extend our multi-dimensional construct of social presence that could be induced by live streaming to examine how different constructs may influence impulse buying in different ways. Finally, there are different modes of live streaming e-commerce: live streaming embedded in e-commerce (e.g., Taobao) and e-commerce embedded in live streaming (e.g., Facebook live). Future studies can investigate the effect of social presence on impulse buying in the two different modes of live streaming e-commerce.

## Figures and Tables

**Table 1:** Constructs and items.

Constructs	Items
Social Presence of the broadcaster [14,20,52]	I can make sense of the streamers’ attitudes by interacting with them in live streaming. There is a sense of human touch when I communicate with the broadcasters in live streaming. Communication with the broadcasters in the live streaming is warm.
Social Presence of the viewer [24,54]	I am aware of other viewers who are interested in the products in live streaming shopping. I am aware of other viewers who share the product’s information in live streaming shopping. I am aware of other viewers who have purchased the products in live streaming shopping.
Social Presence of the live streaming [24,54]	There is a sense of human contact in live streaming shopping. There is a sense of personalness in live streaming shopping. There is a sense of sociability in live streaming shopping
Arousal [22,25]	I feel stimulated when watching live streaming shopping. I feel excited when watching live streaming shopping. I feel surprised when watching live streaming shopping.
Pleasure [22,25]	I feel joyful when watching live streaming shopping. I feel pleasure when watching live streaming shopping. I feel satisfied when watching live streaming shopping.
Impulse buying in live streaming e-commerce [47,55]	While watching live streaming shopping, I often buy things spontaneously. While watching live streaming shopping, I often buy things without thinking. While watching live streaming shopping, I often buy things according to how I feel at the moment.

**Table 2:** Demographic information of respondents.

Characteristics	**Categories**	**Frequency**	**Percentage (%)**
Gender	Male	88	46.56
	Female	101	53.44
Age	18–25	60	31.75
	26–35	110	58.20
	>35	19	10.05
Incomes	<2000	89	47.09
	2000–5000	70	37.04
	>5000	30	15.87
In the past 3 months, how many times do you watch live streaming?			
	1–2	25	13.23
	3–5	83	43.92
	6–10	44	23.28
	>10	37	19.58

**Table 3:** The results of confirmatory factor analysis in the survey.

Constructs	Items	AVE	CR
SPOB	SPOB-1	0.67	0.86
	SPOB-2		
	SPOB-3		
SPOV	SPOV-1	0.55	0.78
	SPOV-2		
	SPOV-3		
SPOLS	SPOLS-1	0.50	0.75
	SPOLS-2		
	SPOLS-3		
Pleasure	Pleasure-1	0.65	0.84
	Pleasure-2		
	Pleasure-3		
Arousal	Arousal-1	0.53	0.77
	Arousal-2		
	Arousal-3		
IB	IB-1	0.65	0.85
	IB-2		
	IB-3		

Note: CR, composite reliabilities; AVE, average variance extracted, SPOB, social presence of the broadcaster; SPOV, social presence of viewers; SPOLS, social presence of the live streaming; IB, impulse buying.

**Table 4:** Inter-construct correlations.

	Mean	SD	SPOB	SPOV	SPOLS	Pleasure	Arousal	IB
SPOB	3.483	0.621	**0.815**					
SPOV	3.332	0.674	0.315	**0.742**				
SPOLS	3.222	0.575	0.401	0.396	**0.703**			
Pleasure	3.448	0.696	0.561	0.303	0.374	**0.803**		
Arousal	3.166	0.693	0.473	0.347	0.537	0.573	**0.727**	
IB	3.541	0.767	0.52	0.388	0.495	0.614	0.633	**0.806**

Note: square root of AVE is shown in bold in the diagonal.

**Table 5:** Result summary of path analysis.

Hypotheses	Coef.	t-Value	Test Result
H1a: SPOB→arousal	0.324 **	3.652	Supported
H1b SPOB→pleasure	0.564 **	6.297	Supported
H2a: SPOV→arousal	0.049	0.526	Not Supported
H2b: SPOV→pleasure	0.022	0.242	Not Supported
H3a: SPOLS→arousal	0.569 **	4.694	Supported
H3b: SPOLS→pleasure	0.231 **	2.254	Supported
H4: Arousal→IB	0.567 **	5.949	Supported
H5: Pleasure→IB	0.383 **	4.646	Supported

Note: ** *p* < 0.01.

**Table 6:** Indirect effect results.

	Total Effect Coef.			Indirect Effects Coef.	Bootstrap 95% CI	Zero Included?
SPOB→IB	0.436 **	0.162 *	SPOB→Arousal→IB	0.106	0.036~0.156	No
			SPOB→Pleasure→IB	0.168	0.054~0.231	No
SPOV→IB	0.184 **	0.116	SPOV→Arousal→IB	0.036	−0.017~0.094	Yes
			SPOV→Pleasure→IB	0.032	−0.012~0.080	Yes
SPOLS→IB	0.385 **	0.176 *	SPOLS→Arousal→IB	0.152	0.052~0.190	No
			SPOLS→Pleasure→IB	0.057	−0.007~0.103	No

Note: ** *p* < 0.01, * *p* < 0.05.

## Data Availability

Not applicable.

## Appendix A

**Table A1:** Item loadings and cross-loadings.

	IB	SPOB	SPOV	Pleasure	SPOLS	Arousal
SPOB-1	0.775	0.176	0.248	0.176	0.154	0.162
SPOB-2	0.806	0.176	0.194	0.071	0.109	0.176
SPOB-3	0.824	0.149	0.161	0.071	0.154	0.071
IB-1	0.233	0.728	0.21	0.219	0.122	0.232
IB-2	0.127	0.824	0.187	0.083	0.191	0.174
IB-3	0.277	0.686	0.299	0.136	0.223	0.189
Pleasure-1	0.355	0.194	0.792	0.004	0.135	0.127
Pleasure-2	0.243	0.336	0.689	0.124	0.04	0.269
Pleasure-3	0.134	0.162	0.825	0.152	0.142	0.126
SPOV-1	0.282	0.01	0.002	0.778	0.048	0.194
SPOV-2	0.033	0.248	0.054	0.811	0.194	−0.027
SPOV-3	0.001	0.084	0.187	0.802	0.164	0.091
SPOLS-1	0.119	0.267	0.07	0.182	0.696	0.183
SPOLS-2	0.172	0.127	0.117	0.208	0.743	0.099
SPOLS-3	0.104	0.073	0.101	0.049	0.813	0.173
Arousal-1	0.063	0.276	0.33	0.182	0.261	0.607
Arousal-2	0.11	0.372	0.222	0.027	0.246	0.639
Arousal-3	0.261	0.115	0.092	0.119	0.151	0.825
